# Formal reasoning on qualitative models of coinfection of HIV and Tuberculosis and HAART therapy

**DOI:** 10.1186/1471-2105-11-S1-S67

**Published:** 2010-01-18

**Authors:** Anil Sorathiya, Andrea Bracciali, Pietro Liò

**Affiliations:** 1 Computer Laboratory, Cambridge University, William Gates Building,15 JJ Thomson Avenue, Cambridge CB3 0FD, UK; 2 Computer Science Department, University of Pisa, Largo Bruno Pontecorvo, 3, Pisa 56127, ITALY

## Abstract

**Background:**

Several diseases, many of which nowadays pandemic, consist of multifactorial pathologies. Paradigmatic examples come from the immune response to pathogens, in which cases the effects of different infections combine together, yielding complex mutual feedback, often a positive one that boosts infection progression in a scenario that can easily become lethal. HIV is one such infection, which weakens the immune system favouring the insurgence of opportunistic infections, amongst which Tuberculosis (TB). The treatment with antiretroviral therapies has shown effective in reducing mortality.

An in-depth understanding of complex systems, like the one consisting of HIV, TB and related therapies, is an open great challenge, on the boundaries of bioinformatics, computational and systems biology.

**Results:**

We present a simplified formalisation of the highly dynamic system consisting of HIV, TB and related therapies, at the cellular level. The progression of the disease (AIDS) depends hence on interactions between viruses, cells, chemokines, the high mutation rate of viruses, the immune response of individuals and the interaction between drugs and infection dynamics.

We first discuss a deterministic model of dual infection (HIV and TB) which is able to capture the long-term dynamics of CD4 T cells, viruses and Tumour Necrosis Factor (TNF). We contrast this model with a stochastic approach which captures intrinsic fluctuations of the biological processes. Furthermore, we also integrate automated reasoning techniques, i.e. probabilistic model checking, in our formal analysis. Beyond numerical simulations, model checking allows general properties (effectiveness of anti-HIV therapies) to be verified against the models by means of an automated procedure. Our work stresses the growing importance and flexibility of model checking techniques in bioinformatics.

In this paper we *i) *describe HIV as a complex case of infectious diseases; *ii) *provide a number of different formal descriptions that suitably account for aspects of interests; *iii) *suggest that the integration of different models together with automated reasoning techniques can improve the understanding of infections and therapies through formal analysis methodologies.

**Conclusion:**

We argue that the described methodology suitably supports the study of viral infections in a formal, automated and expressive manner. We envisage a long-term contribution of this kind of approaches to clinical Bioinformatics and Translational Medicine.

## Background

Human diseases result from abnormalities in an extremely complex system of molecular processes that are often caused by viral or bacterial infections. In these pathological processes, virtually no molecular entity acts in isolation and complexity is caused by the vast amount of dependencies between molecular and phenotype features. The key player of the human survival is the immune system which is a complex system that can be described as a large network of dynamical agents (cells and signalling molecules). A great challenge for contemporary molecular medicine is the modelling, description and ultimately the comprehension of the multistep and multiscale nature of the immune response to pathogens. An even greater challenge is when viral and bacterial infections occur together in the same patient. TB, which is caused by Mycobacterium tuberculosis, is the most frequent co-infection in patients infected by the human immunodeficiency virus type 1 (HIV-1). TB makes more complicated the development of effective therapies.

Efforts for gaining further insights into the pathological mechanisms and novel therapeutic targets benefit from the integration of genomic, proteomic, metabolomic and environmental information. In this work we follow an integrated approach that relies on several sources, such as phylogeny information, traditionally explored within Bioinformatics, differential equations and stochastic modelling, both largely used in Systems Biology, and a formal reasoning technique, viz. Model Checking, developed in Theoretical Computer Science to assess properties of complex computational systems. Furthermore, the Bioinformatics approach in the study of viral dynamics has also focused on identifying variable regions in genomes and pathogenic islands in bacteria [1]. These integrated frameworks appear to be effective for the research in the biomedical problems.

Mathematical and computer science approaches have shown to be more effective in dissecting the network connectivity of cellular circuits and the corresponding dynamical characteristics. The mathematical description of the variation of biomolecular concentrations as a set of ODEs offers quantitative basis for predicting the behaviour and evolution of the system and for testing non-linearities. An alternative approach is to use stochastic simulation via the Gillespie algorithm which provides an exact algorithmic solution of a set of reactions and a meaningful way to consider the noise [2]. The use of mathematical models in immunology has been very successful and has represented an insightful and essential complement to in vivo and in vitro experimental design and interpretation. Kinetic Model suggests that HIV-1 in vivo is continuous and highly productive and that leads CD4 T cells count low [3]. Nowak and May has proposed a simple model on HIV dynamics by considering population of non-infected T cells, infected T cells and viral population [4]. Perelson has proposed mathematical models on interaction of HIV virus and T cells dynamic by considering one more variable (un-infectious viruses) [5]. The HIV quasi-species models have been inspired from the molecular quasi-species model in chemistry [6,7]. Indeed mathematical models of HIV dynamics have proven valuable in understanding the mechanisms of many of the observed features of the progression of the HIV infection, see for example [3,6-13]. In this paper we model coinfection of HIV and TB and the effect of HAART therapy on the model dynamics.

Recently, the observation that biological systems often exhibit interactive and concurrent behavior, similarly to computational concurrent systems, has led to the adoption of formal methods originally developed for the description and analysis of complex software systems in computer science. This abstraction "cell as computation", similar to the "DNA as string" and "protein as labeled graph" abstractions which have originated bioinformatics, has inspired the adoption of model checking methodologies to validate biological complex systems [14]. The growing success of model checking (see for instance [15-18]) relies in the specification of a biological property of interest which is expressed in a formal language, typically a formula of a suitable logic, and its verification is carried out by a fully automated procedure that returns either a positive response or a counter example [19].

The aim of this paper is to pipeline bioinformatics and quantitative models of infectious processes and anti-HIV therapies, and then show how model checking techniques can contribute to the interpretation of the *in-silico *results obtained from quantitative models of infectious diseases dynamics. Given that viral infections and the functioning of therapies often present stochastic aspects, we introduce a succinct but descriptive stochastic model of HIV infection associated with TB opportunistic infection. Then we extend it by modelling (the effects of) HAART anti-HIV therapy. These models have then been implemented in PRISM, a state of the art probabilistic model checker supporting a logical language [20]. We illustrate, by means of two simple properties, the flavour of the verification made possible by these techniques and how this can contribute to a precise assessment of the information conveyed by the mathematical models.

### The complexity of HIV infection and anti-HIV therapies

Human immunodeficiency virus type 1 (HIV-1) infection is characterised by the progressive loss of CD4 T cells. Anti-HIV therapies act to eradicate or lower the concentration of the virus from the body and replenish the CD4 T cells reservoir. Infection by most strains of HIV requires interaction with CD4 T cells and a chemokine receptor, either CXCR4 or CCR5. Viral strains often use CCR5 during early stages of HIV-1 and then switches to CXCR4 to enter into the cells. This switch emerges in more than 50% of patients [21,22] and it has been linked with progression to AIDS because of an increased virulence through the formation of cell syncytia and the stimulation of the cellular factor called Tumour Necrosis Factor (TNF) which inhibits the replication of R5 HIV strains while has no effect on X4 HIV [23,24].

It takes on an average 10 years to get infected by AIDS after having HIV infection. Some patients died within 2 years after getting infected by HIV, while others remained free of AIDS for more than 15 years. The within-patient evolutionary process of viral sequence mutations during HIV infection has suggested improvements in anti-HIV therapies. Anti-HIV drugs are most effective when taken in a combination of three or more at the same time. This is called combination therapy or HAART (Highly Active Antiretroviral Therapy). Physicians recommend starting the therapies if you are ill because of HIV, or if your CD4 T cells count if low (below 200 cells per microL). HAART combinations usually include two drugs that are nucleoside analogues, and one protease inhibitor. The nucleoside analogues drugs result in targeting the viral reverse transcriptase which codes the viral RNA into the DNA that can be integrated into human cells, so transforming the cell into a factory for building blocks of the virus. The protease inhibitor acts as preventing an infected cell from producing new infectious virus particles.

### Bioinformatic links between HIV and TB

**Figure 1:**
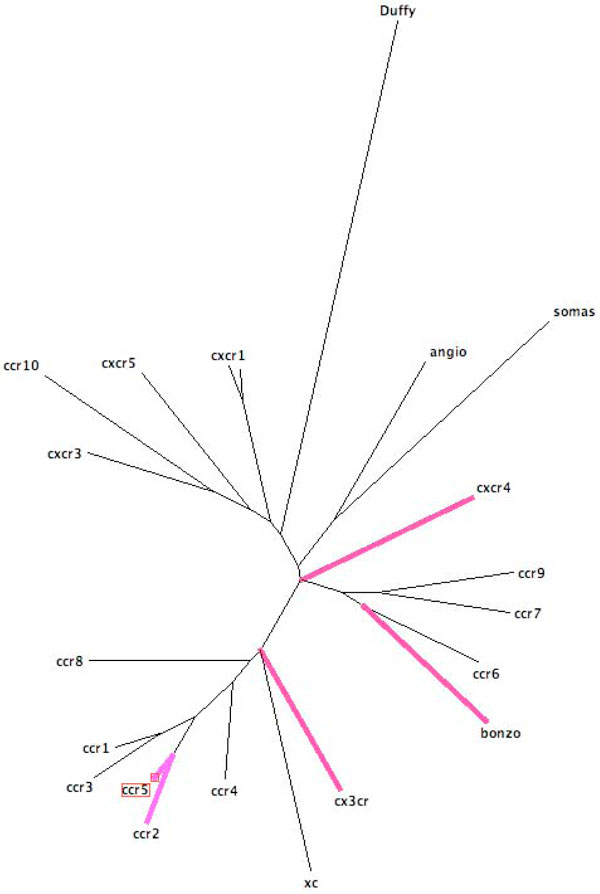
**The maximum likelihood phylogeny under the JTT model of evolution for a set of chemokine receptors amino acid sequences**.

Chemokines provide the key link between HIV and TB infection. Resistance to HIV infection has been found to be related to the following mutations. Delta32 CCR5, 190G CCR2 and 744A CX3CR1 and CCL3L1 [25]. The chemokine receptor CXCR3 can exhibit weak coreceptor function for several human immunodeficiency virus, both HIV-1 and HIV-2 strains and clinical isolates [26]. Gene expression data analysis of HIV infected macrophages showed large changes in beta-chemokines and RANTES (CCL5) [27]. The TB infection is known to produce larger effects than HIV infection on the chemokine networks. TB is associated with excess monocyte chemoattractant protein (MCP)-1 and Tumour Necrosis Factor (TNF)-alpha activity in situ, both are implicated in transcriptional activation of HIV-1 [28]. It is also important to mention significant elevation of the chemokines CCL3, CCL4 and CCL8 [29]. Gene expression data analysis of TB infected macrophages versus uninfected, showed upregulation of the following genes. interleukin-1 beta and interleukin-8, macrophage inflammatory protein-1 alpha, growth-related oncogene-beta, epithelial cell-derived neutrophil-activating peptide-78, macrophage-derived chemokine [30]. The most important mechanism through which TB enhances HIV-1 replication and the progression to AIDS in dually infected patients is the augmentation in expression of TNF-alpha and the HIV-1 noninhibitory beta-chemokine (MCP-1), low presence of HIV-1 inhibitory beta-chemokines (MIP-1 alpha, MIP-1 beta, and RANTES). We have collected a set of human aminoacid sequences of all the chemokine receptors known to chemokines involved in HIV and tuberculosis and a set of control chemokine receptors. We have built a phylogenetic tree that describes the statistical relationship between those chemokine receptors (see Figure 1). The tree was generated using maximum likelihood on an alignment of the sequences of the external regions of the protein loops. In violet the chemokines receptors disrupted by both TB and HIV; in red those disrupted by HIV. Note that the switch between CCR5 and CXCR4 may involve other chemokine receptors. This may suggest to concentrate some efforts on therapies by intervening by blocking other chemokine receptors disfunction.

The disruption caused by the dual infection (HIV and TB) focuses on RANTES which blocks CCR5 and whose expression is upregulated by TNF. Therefore we incorporated TNF in our deterministic model to predict the potential effect of HAART on coinfection of HIV and TB. That evidence would explain the increase in CXCR4 usage with the increase of TNF concentration [31]. The relationship between HIV and TB seems to be very profound since quasispecies of both HIV and TB influence each other differently [32,33]. Our phylogenetic results suggest that both diseases act synergically and not simply additively to alter the chemokine network.

## Results and Discussion

Although differential equation models have long been used for the immune system and viral infection modelling, they focus on the average behavior of large populations of perfectly mixed, identical individuals. An improved realism is perhaps provided by stochastic simulations, which however are computationally intensive. Given that differential equations and stochastic descriptions have important pros and cons and a good degree of complementarity, a framework based on the implementation of both approaches, although time and resources expensive, appears advisable. Stochastic models, then, support the well developed probabilistic model checking analysis. We have extended a model firstly presented in [7]. We have incorporated the Cytotoxic T Lymphocytes (CTLs) response and the dynamics of TB opportunistic infection so as to analyse the co-infection of HIV strains and another disease associated with it.

Furthermore, we have introduced an abstract representation of the HAART therapy treatment by altering the model's parameters that rule the dynamics of our model according to the known effects of the treatment. We are presenting results of the analysis of the HIV and the opportunistic TB infection dynamics and an associated therapy through differential equations, stochastic modelling and formal reasoning techniques.

### HIV and the opportunistic TB infection

**Figure 2:**
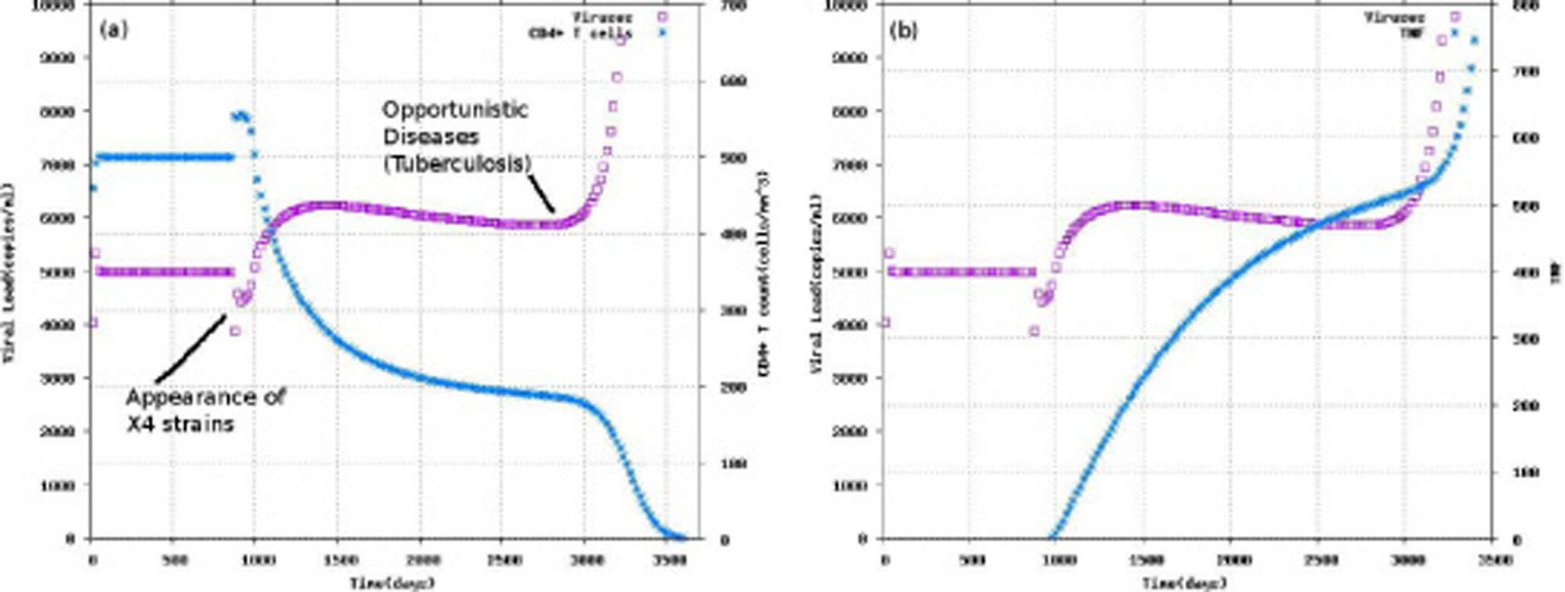
**Viral load, CD4 T and TNF over the course of time**. (a) Time evolution of viral load and CD4 where mutation of V5 leads to V4 at around day t = 900 and opportunistic diseases (TB) appears at around day t = 2900 (b) dynamics of viral load and TNF over time.

Experiments carried out by means the deterministic model are reported in Figure 2. Details about the construction of the model are reported in the Methods section. For the sake of space we do not show the sensitivity analysis of the deterministic model. Experiments with the model are able to faithfully reproduce well known curves of the virus and CD4 T cells dynamics in absence of TB. The introduction of TB provides a novel description of the dynamics occurring with this infection where viral load increases suddenly in the last stage of HIV-1 (AIDS) because of opportunistic diseases (TB) (see [33]). In particular, Figure 2(a) shows the behaviour of CD4 T cells during the switch between R5 and X4 strains. The general dynamics of CD4 T cells and viral load can be observed. In the latest stage of HIV-1 infection (opportunistic diseases stage) a sudden increase of the viral load can be observed, which lead to AIDS. The increase of TNF against the viral load time course is reported in Figure 2(b). This TNF dynamics provide a clear explanation of the quick growth of viruses when TB infection occurs. TB and X4 strain of HIV stimulate TNF production which, in turn, help them to progress at faster rate.

### Modelling HAART therapy against HIV and TB

**Figure 3:**
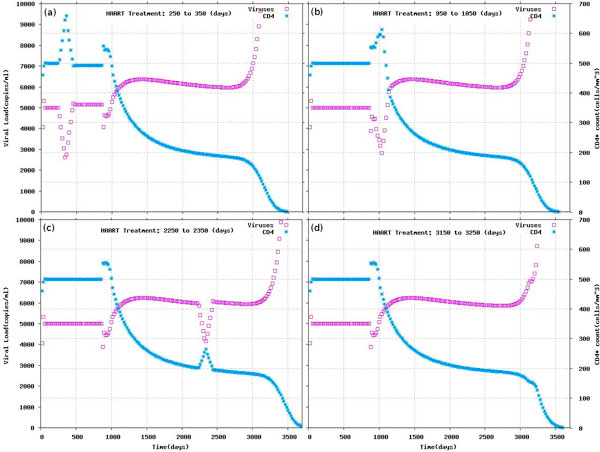
**Experiments with HAART therapy**. Therapy start from (a) day t = 250 to day t = 350 (b) day t = 950 to day t = 1050 (c) day t = 2250 to day t = 2350 and (d) day t = 3150 to day t = 3250.

The model of HIV and TB co-infection can easily be extended to embrace the effect of a common anti-HIV treatment such as the HAART. Drug therapies such as HAART and Maraviroc, although with different mechanisms, result in decreasing the number of viruses and the death of infected cells. Exploiting the expressivity of the treatment, we model HAART in terms of its effects. For the sake of simplicity this can be done by changing the virus replication rate (*π*, see Methods), reflecting a decreased replication rate and budding from the infected cell. Simulation results are reported in Figure 3. Plots (a) and (b) show the effect of the HAART therapy delivered before and after the appearance of the X4 strain, respectively and before (c) and after (d) the TB infection, respectively. Clearly, both the appearance of X4 and the occurrence of TB infection accelerate the decrease of CD4 T cells which the HAART is able to stop but not to completely reverse. An early start promulgated HAART treatment will change the probability of arising of X4 (this results was obtained by Sguanci et al). Here we show that short HAART treatments have small effect if administrated when CD4 T cells count is above 200 and they have even smaller effect when CD4 T cells counts are below 200.

### A stochastic description of HAART, HIV and TB dynamics

Many natural and biological phenomena are intrinsically stochastic and discrete, and they can not always be properly described by means of a deterministic and continuous description. For instance, systemic emergent properties can be sensitive to the local presence of minimal (integer) quantities of molecules [34]. Roughly speaking, a stochastic model associates a probability to each state transition of the modelled system, expressed as a rate associated to the transition. Associated probability distributions are typically memoryless, and hence the overall system behaviour can be interpreted as a Markov Chain. Often, models are used to simulate possible evolutions of the system from initial conditions, [35].

Starting from the deterministic model of HIV, TB and HAART, we have determined a corresponding stochastic model. This has been done via standard transformation from deterministic rates into stochastic ones, according to a fixed reference volume of the model (see, e.g., [36]).

**Figure 4:**
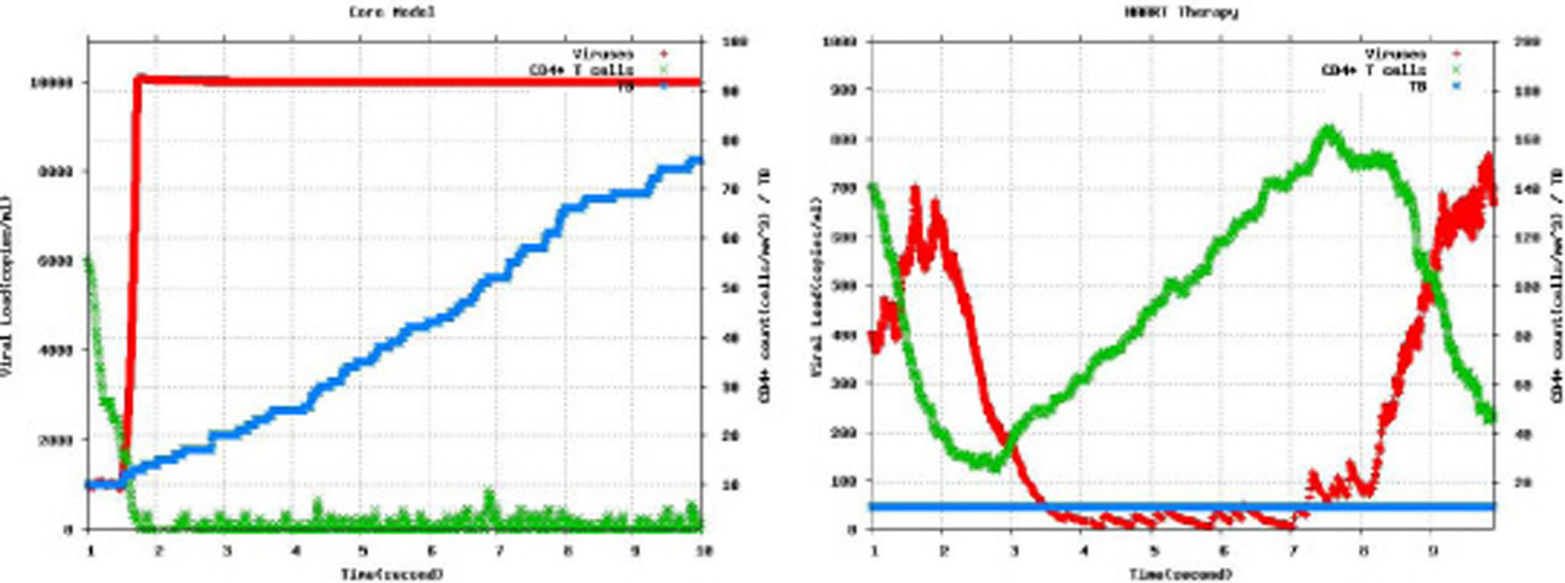
**A limited fragment of the time course of Viruses, CD4 T cells and TB bacteria in the case of (a) no treatment and (b) treatment with HAART**.

Simulation results are reported in Figure 4 about the dynamics observed for a specific possible evolution of the system (chosen according to the probability distribution determined by the model). From Figure 4 (left) it emerges that CD4 T cells, representative of the immune system health, go quickly down in the massive presence of the *X*4 viral strain. As soon as CD4 T is below a threshold limit, 20 units on the scale of this model, TB develops and the viral load saturates. This reproduces, on a small scale, the general time course at individual level, like the one reported in Figure 2. In Figure 4 (right) a simulation under the same initial conditions is shown. In this case, the HAART treatment has been administered starting at a quite early time point (after few tenth of the initial second) and then stopped (at about 2.5 seconds). It can be observed that, even if HIV progression weakens the immune system, it never goes below the threshold, but rather the viral load seems quite well controlled. In the short interval, HAART prevented the development of TB once started before its insurgence. However the viral load upraises after the interruption of the therapy. The stochastic modeling approach shows important advantages, such as the possibility of pipelining with the probabilistic model checking and some limits in the computational intensive requirement. Work in progress will make use of grid computing facilities (EGEE and Cambridge) and cloud facilities.

### Assessing HAART therapy against HIV and TB infection

Observing the quantitative results of the stochastic simulations in Figure 4, one can have an idea about the fact that the HAART therapy is somehow effective. However, we discuss now an example on how it is possible to move from a correct and informative, but somehow empirical, interpretation of data, as the idea above, to a more precise and formal approach by specifying properties of interest as logical formulae. This has the advantage of requiring a definition of all the aspects of interest, of referring to a formal and unambiguous semantics, and of having an automated procedure for verifying formulae (as the one we adopted [20]). Differently, observations rely on the capability of properly interpreting the graphs, which sometimes may appear obscure and difficult to interpret. More details about the methodology can be found in the Methods section.

#### Therapy effects

We focus on two properties that can give a measure of the infection progression.

*i) *we consider the number of infected cells leading to virus replication as a measure of the spread of infection. We are interested in measuring how often this can occur. This can be expressed by a formula like

where *R*{"*i_to_v*"} represents the number of occurrences of an event labelled with *i_to_v*, i.e. the transition from an infected CD4 T cell to virus replication. The question mark says we are interested in determining the number of such possible occurrences in the model dynamics. *C *< 5.0 stands for the time interval;

*ii) *we consider the amount of healthy CD4 T cells, represented by variable *tc*, as a measure of resistance to the virus attack. We are interested in the probability that *tc *is less than the threshold 20. Here ? requires the computation of a probability and *F *[...] *tc *<= 20 is a logic term expressing that the event *tc *<= 20 occurs within the interval [...]:

#### Experimental results

The analysis has been performed using the PRISM model checker (see Methods section). Properties can be validated either by constructing the complete Continuous-time Markov Chain relative to the given model model or by approximating verification through sampling a certain number of possible evolutions. The former can result costly or unfeasible for large models. Adopting the latter we obtained the results reported in Table 1.

**Table 1:** Quantitative results from the automated verification of the effects of HAART therapy. While the number of viral replications due to CD4 T cell infection is comparable with and without HAART treatment for the time interval considered, a much stronger probability of a failure of the immune system, *close to twice as much as*, is observed without the HAART treatment.

	HIV+TB	HIV+TB+HAART
*R*{"*i_to_v*"} = ? [*C *< = 9.0]	250	269

*P *= ? [*F*[5.0, 8.0]*tc *< = 20]	0.885	0.429

Automated verification yields quantitative measures of the investigated properties. While the number of viral replications due to CD4 T cell infection is comparable (but without HAART many other infected cells contribute to virus replication), the probability of a failure of the immune system is much stronger without HAART treatment, *closely to twice as much as*.

## Conclusion

We have illustrated the potential benefits of formal methods and quantitative models when applied to the study of viral infections and therapy assessment within a computational bioinformatics approach. We have done this by presenting experiments on a proof-of-concept scenario regarding HIV infection and the relative TB opportunistic infection. We have adopted an integrated approach, combining deterministic and stochastic techniques and illustrating how properties of interests for the study of viral infections can be formalised in a general purpose logic, as the one supported by PRISM. Our work stresses the growing importance and flexibility of model checking techniques in bioinformatics. Noteworthily, the verification of these properties can precisely characterise the numerical results of simulations, and this can be helpful in comparing and assessing different antiviral therapies. In conclusion, the modelling of HIV infection has two important linked benefits. *i) *it has matured to the stage of allowing us to combine bioinformatics, computational modelling and formal reasoning techniques, as model checking, i.e. it provides a solid bridge between biological systems and the computational objects used to describe them; *ii) *these approaches together capture information that can be valuable in therapy validation suggesting the possibility of moving in the future to realistic cases, i.e. translational medicine and clinical bioinformatics.

## Methods

In this section we provide further details about the construction of the used models and analysis methodologies.

### A deterministic model of HIV strains and TB time evolution

Our work is based on a deterministic model of HIV-1 dynamics, firstly appeared in [7], which takes into account the models developed by Perelson and his followers [3,10,13,37]. These models are well presented and take specific biological reality into account. Our initial model has been extended here by adding two more variables, Cytotoxic T Lymphocytes (CTLs) cells and TB, to capture dynamics of co-infection of HIV and TB.

As standard, we describe the variations of the quantities of the modelled entities as a set of differential equations. We start considering a pool of immature CD4 T cells, represented by the variable *U*, see equation (1). These cells are continuously produced by the thymus at a rate *N*_*U*_, and evolve into differentiated, uninfected T-cells, *T *at a rate *δ*^*U*^. Also, the TNF (*F*) contributes to the clearance of naive T-cells, via , and naive T cells are produced at fix rate *k*_*Z *_due to CTLs (*Z*) response. T cells (*T*) are described by considering their different strains (*T*_*i*_), which we do not detail here (see [7]). Beyond being produced, they can become infected (*I*_*k*_) by interacting with the virus strains *V*_*k *_at rate *β*_*k*_, or die at rate *δ*^*T*^, (equation (5)). Note that the infection parameter *β *is not constant over time, but depends on the distribution of the viral strains R5 and X4 [9]. Infected T cells are cleared out at a fixed rate, *δ*^*I*^, and also due to the action of CTLs with rate , (equation (2)). Equation (6) describes the budding of viruses, i.e. infected cells produce new viruses at rate *π*, and the fact that virus particles may be nonviable or being cleared out at rate *c *by immunoglobulin binding and subsequent engulfments by the macrophages. Next equation (3) describes latent TB (*B*) in the blood which propagates with the rate *α *when T cells goes below a given threshold representing the efficacy of the immune system. The equation (7) describes the TNF (*F*) dependence on X4 strains and on the presence of TB in the blood and the fact that the efficacy of such a factor naturally decay in time. Finally, the response of Cytotoxic T Lymphocyte cells *Z *is as in equation (4): CTLs response depends on number of infected cells (I) and is cleared out at fixed rate *b*. This model is general enough to be used as a framework for fitting real data and simulating superinfection and co-infection patterns.(1)(2)(3)(4)(5)(6)(7)

### The stochastic model

The stochastic model has been derived from the deterministic one. As a simplification we consider only two viral strains: R5 viral strain (*V*5) and X4 viral strain (*V*4), and correspondingly only two kinds of infected cells (I4, I5 and I* for the union of the two). Analogously, quasispecies dynamics revert to *V*5 strains that transform into *V*4 strains (at rate *β*_*k*54_). These two strains are the ones we focus on when observing the time course of infections. As for the deterministic model, we have included sufficient details of interaction among the species to express the properties of interest.

As mentioned, we needed to translate some parameters from deterministic to stochastic ones, accordingly to the reference volume of interest. Other parameters, particularly due to lack of existing data in literature, have been approximated by tuning the model on known macroscopic behaviour. We considered a volume of reaction large enough to contain a statistical reliable number of agents (viruses and cells) and values of extensive quantities are scaled according with the reaction volume considered.

We employ a *population-based *approach where the number of each type of species or cells are modelled, rather than the state of each individual component. Furthermore, also according to the PRISM modeling language we used, we rewrote the equations of the deterministic model in terms of a "reaction-centric" description. dynamics is represented by a set of reaction rules stating the involved entities and the results of interaction. These are reported in Table 2. Reaction (02) stands for the production of T cells from naive T cells, while (04) and (05) represent viral infection. Stochastic parameters are on the right part of the Table. Interestingly, parameters *α *and  vary to reflect the emergent behaviour of TB infection against the efficiency of the immune system. when below to a given threshold, the exponential growth of the HIV virus load appears, caused by the spreading of the infection to other cells and abstractly modelled here by such a set of ad-hoc rules. Initial population of cells in the model is: *U *= 30, *T *= 50, *I *= 30, *Z *= 30, *F *= 0, *V*_4 _= 50 -*V*_5_, *V*_5 _= 50, *B *= 10, and the immune deficiency threshold is set to 20.

**Table 2:** Interactions in the stochastic model. The values of the parameters are from literature referred (for sake of clarity we skip the dimensionality which is the standard reported in literature), see also [42-44], or obtained by tuning of the model.

(01)		*N*_*U *_= 100
(02)		*δ*^*U*^= 0.1
(03)		= 0.00001
(04)		*β*_*k*5 _= 0.0025
(05)		*β*_*k*4 _= 0.0025
(06)		*β*_*k*54 _= 0.025
(07)		*δ*_*T *_= 0.1
(08)		*δ*_*I *_= 0.8
(09)		*δ*_*I *_= 0.8
(10)		*k*_*Z *_= 0.01
(11)		= 0.004
(12)		*π *= 2.5
(13)		*π *= 2.5
(14)		*c*_5 _= 2.5
(15)		*c *= 2.5
(16)		*K*_*F *_= 0.1
(17)		*α *= 2.5 if *T <*20 otherwise 0
(18)		*K*_*B *_= 10
(19)		= 0.00001 if *T *< 20 otherwise 0
(20)		*δ*^*F*^= 0.001
(21)		*b *= 0.2
		where I* stands for *I*4 and *I*5 - two rules.

As far as (the effects of) HAART therapy is concerned, it has been modelled by means of a stochastic triggering event that activates and deactivates the treatment. Activation consists in the modification of interactions (12) and (13), whose rate is downgraded to 1, representing reduced morbidity of the virus, which is one of the main effects of HAART.

#### Probabilistic Model Checking

Being an introduction to probabilistic model checking beyond the scope of this paper, we refer the interested reader to the cited literature and references therein. Other recent works are [15,16,38]. We introduce informally the main concepts by examples.

The information encoded in a stochastic model describes, roughly speaking, the states in which the modelled system can find itself and the associated probability of being in a state at a certain time. For many natural phenomena that follow a memory-less probability distribution this amounts to a (Continuous Time) Markov Chain (CTMC). Furthermore, given a stochastic model, several algorithms to simulate the possible quantitative evolutions of the system have been defined, noticeably the Gillespie's algorithm [2]. State semantics, such as Transition Systems, have been extensively studied and used to precisely describe the behaviour of dynamical systems, like distributed and concurrent computational systems. State semantics may often become hardly manageable due to the exponential explosion of the number of states. However, several efficient and automated analysis techniques have been defined. Logical formulas can represent "homogeneous" sets of states. A formula like

can be used to represent the set of states in which an "*a*-kind-of" transition can occur and the system evolves into a state fulfilling, in turn, the formula *ϕ*. In a sense, the states of the semantics become the model of the formula of interest. The problem of the verification of a formula against a state semantics is known as *Model Checking *[19] and yields either an affirmative answer or a counter example, e.g. a set of states not fulfilling the formula. Most of the times, the model checking procedure is fully automated. Logics [39] and model checking procedures [40] have been defined for probabilistic state semantics, that is *Probabilistic Model Checking*. A typical logic may express formulas like

which, informally speaking, represents all the traces for which *ϕ *holds with probability bigger than *p*. Other examples are *F *>= *t x *> 2, the traces in which *eventually x *> 2 after *t *time units, and *G x *< 10, *globally*, for all the traces, the value of *x *is less than 10.

#### PRISM

**Figure 5:**
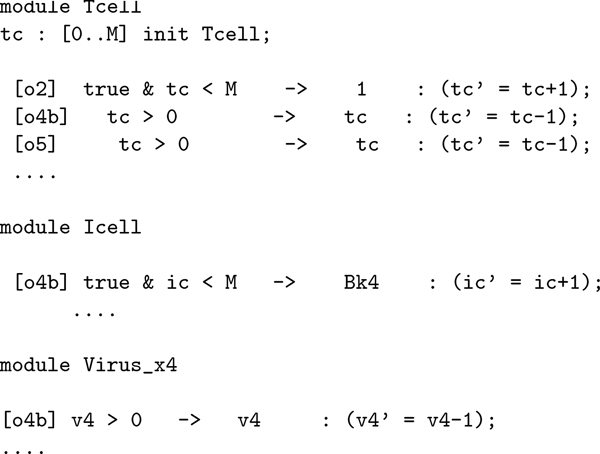
**The implementation of the stochastic model (Table 2) in the PRISM modelling language**.

The PRISM probabilistic model checker [20] is a tool for formal modelling and analysis of systems that exhibit random or probabilistic behaviour. Beyond CTMC, PRISM also supports DTCM and Markov decision processes [41]. PRISM has been chosen as it is one of the reference existing model checker, free and open source. However, its features are not determinant here, and other similar platforms could have been chosen. PRISM models are specified in a formal language that describes the entities present in the model, their behaviour and their quantities. One or more entities can participate synchronously to an event that causes a state change. The state typically consists of the values of the variables of the model. As seen, T cell infection is an event to which virus strains and T cell participate. This event leads to an increased number of infected cells, and less T cells and viruses, which have been combined together. Participation to events has stochastic rates associated. The quantities of entities affect the stochastic dynamics as expected. Figure 5 shows the code for T cell. The variable *tc *keeps trace of the amount of T cells, then two of the possible interactions for T cells are reported, each consisting of a name, an integrity constraint, a rate and an effect: [*o*2] generation of a new T cell, and [*o*5] infection. The [*o*5] interaction is shared with *Virus X4 *and *I cell *(see Table 2). As a consequence of the occurrence of such interaction, the former decreases its amount v4' = v4 -1, which is required to be non-negative, while the amount of the latter is instead increased. It is worth noting how rates are defined so as to respect stochastic semantics. Rates of synchronously executed actions are multiplied to determine the overall rate of the corresponding transition, i.e. the parameter of the negative exponential distribution associated. In the case of [*o*5], the overall rate is correctly determined by multiplying the amount of *T cell *and *Virus X4 *together with the rate *B*_*k*4_. A PRISM model can be used for simulation purposes, and for exact or approximated model checking as illustrated.

## Competing interests

The authors declare that they have no competing interests.

## Authors' contributions

AS and PL authors have studied the deterministic model of infection used in this paper. PL author has investigated the phylogenetic information. AS and AB authors have defined the stochastic version of the model and run in-silico experiments. All authors have worked on experiments results.
